# Therapeutics formulated to target cancer stem cells: Is it in our future?

**DOI:** 10.1186/1475-2867-11-7

**Published:** 2011-03-25

**Authors:** Stephanie Clayton, Shaker A Mousa

**Affiliations:** 1 The Pharmaceutical Research Institute at Albany College of Pharmacy and Health Sciences, One Discovery Drive, Rensselaer, NY, 12144, USA; 2 College of Medicine, King Saud University, Riyadh, 11461, Saudi Arabia

**Keywords:** Cancer Stem Cell, Cancer Stem Cell Like, Molecular Signature, Cell Surface Markers, Microenvironment, Targeted Therapy, Chemoresistance, Cancer Recurrence, Signaling Pathways

## Abstract

With the political, social and financial drives for cancer research, many advances have been made in the treatment of many different cancer types. For example, given the increase in awareness, early detection, and treatment of breast and prostate cancers, we have seen substantial increases in survival rates. Unfortunately there are some realms of cancer that have not seen these substantial advancements, largely due to their rapid progression and the inability to specifically target therapy.

The hypothesis that cancers arise from a small population of cells, called cancer stem cells (CSCs), is gaining more popularity amongst researchers. There are, however, still many skeptics who bring into question the validity of this theory. Many skeptics believe that there is not a specific subset of cells that originate with these characteristics, but that they develop certain features over time making them more resistant to conventional therapy. It is theorized that many of the relapses occurring after remission are due to our inability to destroy the self-renewing CSCs. This central idea, that CSCs are biologically different from all other cancer cells, has directed research towards the development of therapy to target CSCs directly. The major dilemma in targeting therapy in myeloproliferative disorders, malignancies of the central nervous system or malignancies in general, is the inability to target CSCs as opposed to normal stem cells. However, with the recent advances in the identifications of unique molecular signatures for CSCs along with ongoing clinical trials targeting CSCs, it is possible to use targeted nanotechnology-based strategies in the management of different types of cancers.

## Introduction

When discussing potential targets for the treatment of cancer today, the conversation will generally lean towards targeted therapy of cancer stem cells (CSCs). With the identification of potential defining characteristics for CSCs, there have also been more questions raised as to which of these characteristics may make better targets. For many years, research seemed to focus on isolating CSCs by specific identifying markers but the research has seemed to shift towards identifying the way in which these stem cells behave that make them different from bulk tumor cells. Limited efficacy has been seen with the use of cell surface markers in clinical trials; however, there have been recent advances that target other aspects such as signaling pathways or genetic alterations seen particularly in CSCs. The following is a review of what information is out there and what seem to be the most promising paths on this journey to identifying therapeutic targets of self-renewing CSC sub-populations.

### Identifying Characteristic Cell Surface Markers

Identifying CSCs by their outer appearance or cell surface markers has been focused on by many researchers. The concept of identifying CSCs by these markers is a rational one. The challenge in targeting CSCs is identifying which cell surface markers are going to be the distinguishing factors that will make them a suitable target.

One of the biggest discoveries in the identification of cell surface markers involved leukemic stem cells (LSCs). The discovery of CD34+/CD38- as a cell surface marker on AML leukemic cells gave the first indication that there may be distinguishing cell surface markers that would allow for targeting of CSCs[1,2]. With this identification it was determined that only cells that were located in the CD34+/CD38- population of progenitor cells had the capacity to initiate leukemia in NOD-SCID mice when compared with CD34- and CD34+/CD38+ cells [2]. Upon further investigation it was determined that those cells that expressed CD34 on their cell surface also strongly expressed BCRP, a member of the ABC transporters, which play an important role in dug efflux. It has also been found that BCRP is the key player in drug efflux in AML leukemic cells as opposed to P-gP which is common in many other biological systems [3]. Although identification of this subpopulation is an important discovery in terms of narrowing the search for a viable target, it only gives information that LSCs are derived from a subpopulation of immature bone marrow cells. It did also provide researchers with a definition of CSCs for AML. It identified a separate population within AML cells that were able to cause cancer transplantation into NON-SCID mice. For this reason there has been a movement in cancer research to target subpopulations within the CD34+/CD38- subpopulation in order to further target LSCs (Table 1).

**Table 1:** Cancer Stem Cell molecular signatures in different cancer types: Potential for CSC targeting

Target Type	Specific Target	Cancer Type	Use
Cell surface markers	CD34+/CD38-	Acute Myelogenous Leukemia	Identification has allowed for characterization of LSCs. Too broad to use as a target for chemotherapy but is very useful in identification for further characterization.

	CD33+	Acute Myelogenous Leukemia	Gemtuzumab ozogamacin

	C-type lectin like molecule - 1 (CLL-1)	Acute Myelogenous Leukemia	No clinical trials but efficacy seen in vitro and in vivo experimental studies.

Signaling Pathways	PI3K/Akt/mTOR	FDA approved therapy for renal cell carcinoma. Evidence that may be effective in other solid tumors.	Temsirolimus, Everolimus FDA approved for renal cell carcinoma.

	Hedgehog	Evidence in basal cell carcinoma but has been identified as being up-regulated in many cancer types.	Novel GDC-0449

	HMG-CoA reductase	Increase ROS within cells leading to apoptosis, being investigated in many cancers including CML.	Synergistic effect seen when imatinib and simvastatin in CML.

Microenvironment	Mesenchymal stem cells	Evidence in Glioma but being investigated in many solid tumors.	Used in vivo as a target, also being investigated as a drug delivery system.

Another cell surface marker widely used in the study of AML treatment is CD33, given its extensive expression on LSCs. CD33 is an immunoglobulin that is believed to aid in regulation of cellular differentiation [4]. CD33 has been found to be expressed on 80-90% of leukemic cells in those patients suffering from AML. Not only has CD33 been widely used in research but it has also made it as far as FDA approval [3,5]. Anti-CD33 antibodies have become an important aspect of CSC targeted therapy. A therapy, called Gemtuzumab ozogamacin (GO) or Mylotarg, approved by the FDA in 2000, combines calicheamicin (a cytotoxic antibiotic) with an anti-CD33 antibody. Mylotarg has been approved for use in CD33+ AML patients who are 60 years of age or older, who are not candidates for other cytotoxic chemotherapy but are experiencing 1^st ^relapse. Guidelines for the treatment of elderly patients suffering from AML still indicate the use of intensive chemotherapy as first line in those who are in good enough health to receive it [6]. Those who are candidates for treatment with intensive chemotherapy, such as daunorubicin in combination with cytarabine, generally are less than 70 years of age, have a WBC <100 × 10^9^/l and no adverse cytogenic abnormalities or MDR expression. These general characteristics are reiterated in a study by the Southwest Oncology Group that assessed cytogenic and multidrug resistance subgroups in elderly patients who were refractory to standard chemotherapy treatment [7].

A phase I trial, conducted by Sievers et al., first gave insight into the use of Mylotarg in patients with refractory or relapsed AML [8]. This study investigated the effects of what is now Mylotarg on 40 relapsed AML patients, with a median age of 54. Disappearance of leukemia, among the trial participants, was indicated by absence of leukemic blast cells in the peripheral blood with <5% leukemic blasts present in the bone marrow. Further, complete remission was defined by disappearance of disease plus an ANC > 1,500/ul and a platelet count .100 × 10^3^/ul, without transfusions. Results from this trial showed that 8 of the 40 patients (20%) of those treated with GO experienced complete remission [8]. Table 2 illustrates ongoing clinical trials targeting CSC in different cancer types.

**Table 2:** Update on clinical trials for CSC molecular targets

Target	Drug	Cancer	Phase	http://clinicaltrials.gov/	Sponsor
				Identifier	
Notch	MK0752	Breast	I	NCT00106145	Merck
		Pancreatic	I, II	NCT01098344	Cancer Research UK
	RO4929097	Renal cell	II	NCT01141569	University Health Network, Toronto
	PF-03084014	Leukemia	I	NCT00878189	Pfizer
Hedgehog	GDC-0449	Solid tumors	I	NCT00968981	Genentech
		Colorectal	II	NCT00636610	Genentech
	PF04449913	Hematologic	I	NCT00953758	Pfizer
	BMS833923	Basal cell	I	NCT00670189	Bristol-Myers Squibb
	LDE225	Medulloblastoma	I	NCT00880308	Novartis

Modified from (Ref. [44])

In an interim report for a study comprised of 3 open-labeled, phase II, multicenter trials, performed in 2001, the safety and efficacy of Mylotarg treatment in AML patients experiencing first relapse, was determined [9]. There were 2 types of responses evaluated during this study. A complete response was defined by leukemic blasts absent from the peripheral blood, <5% blasts bone marrow aspirate or biopsy, peripheral blood counts with hemoglobin levels of 9 g/dL or greater, ANC ≥ 1,500/ul and platelet count ≥ 100,00/ul and RBC transfusion independence for 2 weeks and platelet transfusion independence of at least 1 week. There was also a subset of those evaluated who experienced complete response with the exception of full recovery of platelet counts before they required the next treatment(CR_p_) [9]. The number of people that experienced these responses was combined to determine an overall response rate for the study. This study, composed of 142 CD33+ AML patients with a median age of 61 years, showed that there was an overall response rate of 30% with a median time to response of 60 days. It was also indicated that the median overall survival was 5.9 months [9]. The final report for this study, published in 2005, indicated similar results [10]. The final report showed that among the 277 patients treated with GO, there was a 26% response rate with a median overall survival of 4.9 months.

According to a new phase III trial that studied the effect of GO on AML patients who were in remission, there was no increase in survival rates among those who used GO when compared to no treatment post remission [11]. Patients in this study were composed of those patients who had experienced complete remission who were then offered 3 cycles of GO or no further treatment. The purpose of this study was to investigate whether treatment with GO post remission may be instrumental in preventing relapse among AML patients. This study included 232 patients who were randomized to either the treatment with GO group or the no treatment group (113 patients in GO arm and 119 patients in no treatment arm). Among these patients there were 2 types of induction chemotherapy used in order to obtain the complete remission. These treatments included induction therapy with 45 mg/m^2 ^dose schedule of daunorubicin (days 1, 2, 3) and cytarabine 200 mg/m^2 ^(days 1-7) or induction therapy with daunorubicin 90 mg/m^2^. Among the treatment and no further treatment arms, there was no statistically significant difference in the amount of patients who used either therapy [11]. As stated, this study showed no statistically significant difference in survival rates between these 2 groups. This study also provided a more lucid adverse effect profile for Mylotarg. Among the adverse effects of fever, sepsis and hepatic and gastrointestinal toxicities, there was also a strong indication of hematologic toxicity, commonly seen in the form of cytopenias [11]. This might not seem all that surprising as they are the all too familiar adverse effects associated with the majority of chemotherapy; however, this is evidence that the common concentration of CD33 on normal cells needs further investigation.

There may be many reasons why there was no statistically significant difference in survival rates seen among those who were treated with GO and those who received no treatment post remission. One reason may have been that there was a decrease in the expression of CD33 on CSCs but another reason may have been due to efflux mechanisms associated with CSCs. A brief report on the phase II trials mentioned previously [9,10] showed a potential correlation between response to GO therapy and P-gP activity [12]. This report evaluated all of the patients who were treated with GO and compared the responders (CR and CR_p_) to non-responders. Results show that there may be an increase in P-gp activity and a decrease in CD33 expression in those who did not respond to GO therapy [12]. Further studies have been done to determine what LSC characteristics are associated with an increased sensitivity to GO. An in vitro analysis of chemo-sensitivity of LSCs, performed by Jawad et al., indicated a correlation between high CD33 expression, P-gp-negative status and low % leukemic stem and progenitor cells and GO sensitivity [13].

One cell surface marker that seems to be gaining popularity is C-type lectin-like molecule or CLL-1. CLL-1 is a type II, transmembrane glycoprotein that has become the subject of interest in the targeted treatment of LSCs [14]. The identification of CLL-1+ cells within the CD34+/CD38- subpopulation has lead to not only a potential target for therapy but also as a marker in diagnosis and prognosis [15]. One of the hardest parts of finding a cell surface marker as a target in the treatment of LSCs is being able to identify one that is present in all cases of that cancer but at the same time not present on normal cells. For this reason, an in vitro study that identified that CLL-1 is present on AML CD34+/CD38- cells but is not present on normal bone marrow CD34+/CD38- cells is an important find in terms of potential targets for AML [15]. This study included leukemic cell samples from 89 patients who underwent Fluorescence-activated cell sorting (FACS) in order to obtain leukemic cells that were CD34+/CD38-/CLL-1+. When taking into consideration different types of AML based on the French-American-British classification, it was determined that CLL-1+ was present on all classes of FAB (M0-M6). From this study it was determined that the expression of CLL-1 varies vastly between samples and seems to have no correlation with the different FAB classes. This in turn indicates that they have found no significant correlation between expression of CLL-1 and potential prognostic factors. It may be important to note that normal bone marrow samples of CD34+/CD38+ progenitor cells were partly CLL-1+ as well [15]. This is important because it means that care should be taken before specifically targeting CLL-1 cells rather than targeting these cells within a subpopulation. From this study it was also determined that NOD/SCID mice transfected with CD34+/CD38-/CLL-1+ cells were able to produce AML blasts cells that were CLL-1+. Although this does not indicate that CLL-1 is required for transfection of AML because it was not compared to CD34+CD38-/CLL-1- cells, it does offer a potential target in terms of CSCs. The potential as a target comes from the fact that none of the CD34+/CD38- cells from normal bone marrow expressed CLL-1 whereas all of the AML CD34+/CD38- cells expressed CLL-1 in this study. As indicated in a 2010 review article, expression of CLL-1 has been detected on as high as 92% of AML cases [4]. This can be truly important if identification of a cell surface marker that can be targeted in all types of AML is possible.

Given the heterogenic response to GO among AML patients experiencing remission, further studies should be done to determine what targets could be added to this treatment to increase the response rates. Also further research should be considered in finding an alternative target such as CLL-1 given the fact that studies have shown that chemotherapy resistant lines of AML, such as K562, are CD33- [16]. With the adverse effect profile noted in recent clinical studies, it is likely that CD33 may be fairly extensive on normal humans stem cells (HSCs) and for this reason should be re-evaluated as a potential target [11]. Although CD33+ cells are predominantly located within the CD34+CD38- subpopulation, their presence may not be a defining characteristic of LSCs.

As mentioned previously, a viable target for AML is CLL-1 as a cell surface maker. There have been advances in the identification and characterization of CLL-1 and its relationship to AML. Given its almost exclusive expression on AML blast cells and its expression seen in all types of AML, it may be a viable target although there is not much evidence as of yet to indicate if there are any therapeutic uses in targeting AML CSCs. The study of LSCs may benefit from combining anti-CLL-1 antibodies with conventional AML chemotherapy such as daunorubicin. It will only be with studies like these that current implications of CLL-1 targeting in AML can be verified.

Different formulations for conventional chemotherapy without specific targets have been attempted but indicate no benefit over the conventional treatment. One such study that indicates this is the comparison of daunorubicin and liposomal daunorubicin in older patients experiencing complete remission of AML. This study resulted in no statistically significant differences in toxicity or response among the 2 different treatment groups. Results of this study further indicate the need for therapies that are targeted to more specific cells [17].

### Targeting of Signaling Pathways

Another means of targeting CSCs has been by signaling pathways that seem to be up-regulated or specific to the functionality of stem cells. Just as a friend can be identified by superficial characteristics, they can also be identified by the way that they speak or the way that they behave. The fact that many cancers can share the same up-regulation of certain pathways also makes targeting of stem cell signaling pathways a good option. Some of the pathway targeting that seems to show some potential is the PI3K/Akt and Hedgehog pathways.

The activation or up-regulation of this pathway has been associated with activation of survival and proliferative mechanisms utilized by malignant cells [18]. The entire PI3K/Akt/mTOR pathway is a long cascade of phosphorylative reactions; however, there are a few very important key players that have been studied as potential targets due to their implications in tumorogenic activities. The play that is farthest upstream is Phosphatidylinositol 3-kinase (PI3K) which is a heterodimeric lipid kinase that plays a key role in part of the PI3K/Akt/mTOR pathway. PI3Ks are divided into classes of I, II or III and then further into subclasses. Class I PI3K works to phsophorylate the 3'-OH group of inositol lipids of which gives rise to, among many products, Akt [19]. Activating mutations in the gene encoding for p110 (PIK3CA), a catalytic subunit of Class IA PI3Ks, have been found in many different types of human cancer [20]. Akt2, one of three Akt isoforms, is a protein kinase which, when activated, plays a critical role in tumor metastasis and invasion.

Downstream of Akt is the mammalian target of rapamycin (mTOR). mTOR is so named for its ability to be inhibited by rapamycin, also known as sirolimus, which is an immunosuppressant used to prevent renal transplant rejection [21]. It is critical for the production of mRNA that is vital for cancer cell growth [18]. mTOR is an enzyme that functions as a protein kinase and is involved in production of many products used for cell proliferation, survival and angiogenesis, for example VEGF [20]. mTOR can also be classified by different complexes that can be formed including mTOR complex 1 (mTORC1) and mTOR complex 2 (mTORC2), both which play critical roles in cell survival [20]. Each portion of the pathway is indicated in different functions for enhancement of tumorogenic effects but overall, activation of this pathway leads to decreased apoptosis and autophagy and an increase in translation, cell growth, ribosome biogenesis, metabolism and proliferation in cancer cells [20]. In a recent in vitro study performed by Bleau et al., identified the PTEN/PI3K/Akt pathway as playing a key role in characteristic features of glioma side-population cells [22]. Side populations have been identified as playing a key role in the identification of CSCs. The discovery of side populations came from staining of bone marrow cells with Hoechst 33342 vital dye and discovering that there was a small population of cells that were not stained but also expressed certain CSC surface markers previously identified [23,24]. Side populations are believed to have an efflux mechanism that allows the Hoechst dye to be expelled from the cells. This efflux mechanism is also believed to play a role in the multiple drug resistance effects associated with CSCs. The side populations in the study were identified using the same method of staining with Hoechst dye and were then evaluated for a correlation with the PTEN/PI3K/Akt pathway. This correlation was determined when the amount of SP cells in PDGF-induced glioma (a well-characterized form of glioma) of PTEN intact mice was compared to the amount of side population cells in PTEN-deficient mice. The loss of PTEN resulted in a doubling in the amount of side population cells. The PTEN-deficient cells contained 33.1% sp cells while the cell with PTEN intact exhibited 15.5% sp cells. This can confer that up-regulation of the PI3K/Akt pathway can be implicated in the survival and proliferation of CSCs. In order to determine the role of this pathway on resistance the PTEN-depleted and PTEN-intact cells were incubated with mitoxantrone both before and after incubation with a PI3K inhibitor (LY294002, 20ྒྷM). As suspected the PTEN-deleted cells resulted in an increased resistance to mitoxantrone and incubation with LY294002 resulted in a significant decrease in both the PTEN-intact (3.6 ± 0.4%-fold) and in the PTEN-deleted (3.0 ± 0.7%-fold) tumors. Of note, when the side population cells were tested with mTOR and Akt inhibitors, mTOR inhibition resulted in a limited effect on termination of side population cells while Akt inhibitors resulted in complete inability of the transporter to cause efflux of mitoxantrone [22]. Indications that the PI3K pathway is constitutively active in 30-40% of human cancers make it a good potential target that may yield benefits in the entire field of cancer rather than a target that will show results in a very specific cancer type [18].

Investigations into the PI3K/Akt/mTOR pathway have also shown some potential for targeting CSCs [20,22,25]. Integrin linked kinase (ILK) is also involved in phosphorylation of Akt and is over-expressed in many malignancies including AML blast cells[26]. One of the hardest parts of targeting cancers is being able to target cells when they are quiescent. Interestingly, there is an over-expression of ILK during this phase which may play a part in the survival of cells or prevention of apoptosis[26]. Based on this evidence, research was performed to determine the effect of using an ILK inhibitor along with chemotherapy to target active cells as well as those that were quiescent. When a novel ILK inhibitor (QLT0267) was administered in vitro to cultured AML cells, with either Ara-C or Daunorubicin, there was in, most cases, a synergistic or additive effect. In 2 out of 10 cases there was, however, an antagonistic effect[26].

One of the targets in this pathway with the most outcomes is a class called rapamycin inhibitors. This class of inhibitors works on the mammalian target of rapamycin or mTOR portion of the pathway [27]. This pathway is found farthest downstream in the PI3K/Akt/mTOR pathway and seems to have the most evidence so far, showing effectiveness in the treatment of renal cell carcinoma. Although this has the most clinical evidence to date, there are other indications, as mentioned previously, that other parts of the pathways may have more benefit in targeted cancer therapy [22,27]. Two rapamycin analogs, temsirolimus and everolimus, have received FDA approval for use in the treatment of renal cell carcinoma. Rapamycin as well as theses rapamycin analogs do not exhibit their effects by direct binding to the catalytic site of mTORC1 but rather bind FK506 (mTORC1 immunophilin). Binding of this complex to mTORC1 results in inhibition of signaling events further downstream [20]. There have also been many clinical trials performed as well as many clinical trials in process that investigate monotherapy or combination therapy with both temsirolimus and everolimus in other types of cancers[28-30]. Results of these trials indicate some efficacy in terms of increasing progression free survival rates, however, results at the end of the studies were generally still dismal overall. In the clinical trial comparing temsirolimus and an investigator's choice therapy, there was a statistically significant increase in survival rates in patients suffering from refractory or relapsed mantle cell lymphoma (MCL). Investigator's choice therapy was a single dose of gemcitabine, fludarabine, chlorambucil, cladribine, etoposide, cyclophosphamide, thalidomide, vinblastine, alemtuzumab or lenalidomide. The median progression free survival rates for high dose temsirolimus, low dose temsirolimus and investigator's choice therapy were 4.8, 3.4 and 1.9 months respectively [28]. While these results are statistically significant, they do not seem to do much for the overall clinical outcomes. In a recent trial, the response to treatment with everolimus 10 mg in those suffering from Waldenstrom Macroglobulinemia was studied. Waldenstrom Macroglobulinemia is a B-cell lymphoproliferative disorder. Each cycle was defined as 4 weeks at which time a CBC was performed to evaluate ANC, platelet count and presence of grade 3 or 4 hematological toxicities. If patients had no sign of toxicities they were continued on treatment with 10 mg daily. If the patients experienced toxicities, treatment was stopped until signs resolved at which time they were treated in a stepwise fashion up to 5 mg everolimus daily. This clinical trial studying everolimus treatment, in 51 patients, showed no complete response, 42% with partial remission and 28% with a minimal response. When the article was published 14% of patients had died and 26% of patients had experienced disease progression [30]. Disease progression was defined as a 25% increase in monoclonal protein from baseline. When comparing toxicities seen between these 2 studies, it seems as though there are some added toxicities associated with treatment, more of which seemed to occur in temsirolimus. There was however a much larger sample size being investigated in treatment with temsirolimus. Upon treatment with everolimus, 56% of patients experienced grade 3 or higher toxicities while patients treated with temsirolimus resulted in grade 3 or higher toxicities in 80-89% of patients depending on dose [28,30].

Phosphatase and tensin homolog (PTEN) is a lipid and protein phosphatase that is drawing much attention in cancer given its tumor suppressing effects which have been negated by genetic alterations [20,22,31]. PTEN inhibits the phosphorylative effects of PI3K by dephosphorylating phosphatidylinositol-3-triphosphate which is a product of PI3K activity [32]. With these genetic alterations, PTEN is inhibited and the PI3K pathway is free to up-regulate resulting in increased cell proliferation and decreased apoptosis. The above mentioned studies and review indicate that PTEN could be another possible step to target in the PI3K pathway.

Another potential target that seems to show some efficacy is the targeting of the Hedgehog (Hh) pathway [33]. The hedgehog pathway is characterized by a few key players including the hedgehog ligand, the Patch (PTCH) transmembrane receptor and the Smoothened (SMO) transmembrane protein. Under normal circumstances, PTCH is an inhibitory cell-surface receptor that acts a tumor-suppressor which acts by inhibiting smoothened, which in turn inhibits further activations of the hedgehog pathway [34].

Alterations and activation of the hedgehog signaling have been shown to play a role in the survival of medulloblastoma, basal cell carcinoma, pancreatic adenocarcinoma and small lung cell carcinoma [35]. Smoothened, a transmembrane protein has been identified as central to the activation of the Hh pathway [35]. A recent study has shown that by genetically modifying mice to make them devoid of the Smoothened allele there is no effect on the survival or maintenance of normal hematopoietic cells. After analyzing the effects on the mice, it was determined that there was no difference in peripheral cells counts and no effect even on repopulation of stem cells after stress [35]. These finding can be very important in that it identifies the Hedgehog as a target that may result in a therapy with decreased side effects.

Although there is limited data available in terms of clinical trials there are many that are in progress to determine the use of hedgehog inhibitors in the treatment of different cancer types. One novel Hh inhibitor, GDC-0449, has been examined in an open label clinical trial [33]. This study investigated the use of GDC-0449 in patients suffering from advanced basal cell carcinoma. A total of 33 patients with advanced basal cell carcinoma, 18 of which had metastatic carcinoma, were treated with GDC-0449. The overall response rate among those with metastases was 50% while the 15 patients with localized disease experienced a 60% response rate [33]. Of note, 2 patients with advanced lesions on the face and head showed a drastic decrease in the size of the lesions. These results can indicate an improvement in the overall quality of life in those experiencing such deforming lesions.

Some new studies are coming forth that are exploring the use of statins for additive treatment during chemotherapy treatment. Statins or HMG-CoA reductase inhibitors are believed to play a role in reactive oxygen species (ROS) activity by leading to increases in NO activity within cells. ROS include superoxide, peroxides and hydroxyl radicals and are known to play important roles in aging and apoptosis among other things. Increases in ROS within the cells cause damage to DNA and protein that at low levels can actually lead to different types of cancers. However, high levels of ROS can be damaging to cancer cells and cause apoptosis [36]. There are beliefs that CSCs utilize ways to avoid these oxidative stresses to increase the rate of survival. One theory is that they act as anaerobic bacteria do, by having low production of mitochondrial oxidative phosphorylation. It is believed that during these hypoxic conditions, there is a large production of ATP, which researchers believe may be due, in part, to synthesis by Acetyl-coA synthetase [36]. HMG-coA reductase produces mevalonate and other products that indicated in the control of cellular functions like cell signaling and cell cycle progression [37]. Researchers have indicated that apoptosis induction by statin can occur by inhibiting HMG-coA reductase but also by increased production of Nitric oxide levels [36,37]. This increase in NO activity may lead to an increase in apoptosis as shown by increased survival among cells pre-treated with an iNOS inhibitor prior to treatment with statins [37]. Many studies have been performed to analyze the use of statins as an adjunct to chemotherapy but one in particular may offer insight to its potential use in the treatment of leukemias. This study, performed by Chen et al., showed the synergistic effect of imatinib and simvastatin in the treatment of CML. This synergistic effect was believed to be due to an increase in ROS levels within the cancer cells leading to apoptosis, indicated by the lack of synergy when a NAC, a ROS scavenger, was added to the simvastatin and imatinib combination [38]. The use of statins for potential synergistic cytotoxic effects is exciting given the ease of administration and its relatively low side effect profile.

Another exciting target against survival mechanisms of CSCs are the efflux mechanisms. The discovery of efflux mechanisms associated with CSCs has primarily come from the study of "side populations." Side populations, as described previously, are present in many types of cancers and are associated with the CSC population. One of the most common targets among these efflux pumps is the P-Glycoprotein (P-gp) pump. A recent study showed great targeting potential using anti-P-gp functionalized oxidized single walled carbon nanotubules (Ap-SWNTs) combined with doxorubicin and its effect on AML K562R cells. K562R cells are shown to be very resistant to chemotherapy and for this reason were a good candidate for this study. From this study it was determined that using Ap-SWNT loaded with doxorubicin extensively decreased cell viability when compared to doxorubicin alone and with other targeting mechanisms. This was an in vitro study performed in culture so it may be beneficial to perform in vivo studies in murine models [39].

Another recent study indicated that the use of cyclosporine may be a candidate for inhibition of P-gp and may have advantages for concomitant use with chemotherapy. This was demonstrated by comparing daunorubicin alone and daunorubicin plus cyclosporine in the K562/ADM strain of AML. Results of this study indicated that after 6 hours of incubation with daunorubicin plus cyclosporine, the sensitivity of the K562/ADM strain approached that of the daunorubicin sensitive K562 strain of AML cells [40].

Overall, if research can reveal the mechanisms that are used by LSCs to avoid apoptosis or increase survival rates, new therapies can be derived that target these mechanisms. Moving forward in the study of survival mechanisms, there seems to be a great value in the study of efflux mechanisms. As with many other cancer treatments, the best results will likely be seen when combining cytotoxic drugs with targets for P-gp efflux mechanisms.

### Targeting the Microenvironment

Along with the increase in research targeting LSCs specifically, there is also an increase in research that will target their lifeline. One key area of research is determining the effect of mesenchymal stem cells on CSCs. Studies have indicated that the presence of mesenchymal stem cells with LSCs leads to a reduction in proliferation and a decrease in apoptosis. One study evaluated the proliferation of leukemic cells that were exposed to a serum deprivation of starvation condition with or without mesenchymal stem cells. There was a very large decrease in the amount of cells produced in the plates that co-cultured mesenchymal stem cells and K562 (leukemic strain) cells [41]. This leads to the belief that mesenchymal stem cells can help force cancerous stem cells into a quiescent state making them less susceptible to conventional cancer treatments that target actively proliferating cancer cells. This decrease in apoptosis is believed to be contributed to up-regulation of PI3K-Akt pathway [42].

Although mesenchymal stem cells have been identified as a life-line for LSCs, recent studies have identified them as potential drug delivery systems. Some therapies that have been developed in the treatment of brain tumors such as glioma, have shown effective therapy when injected intra-tumorally. An example of such treatment is the monoclonal Ab 806 which targets the ΔEGFR, an epidermal growth factor shown to be up-regulated in cancers such as glioma, breast and lung. Treatment of mice with xenografted gliomas with mAb 806 had a decrease in tumor growth as well as a 61.5% increase in median survival rates compared to those who were not [43]. These results, however, were transient and resulted in relapse and increased growth of the tumor. The theory behind this relapse was that the treatment was not delivered throughout the tumor [43]. In order to target malignant diseases, there is a need for systemically administered therapies to be able to home in on their intended targets. Recent studies have showed results in the treatment of glioma that may offer insight into the treatment of malignant diseases. One such study shows that human mesenchymal stem cells (hMSC), genetically modified to secrete single-antibody fragments that have specific high affinity binding for EGFRvII (scFvEGFRvIII), can be identified in much higher concentrations within the brain tumor cells than other organs [41]. Although this study was developed to elucidate treatment options for glioma, this could likely be an optimal area of research for treatment of many different cancers. If mesenchymal stem cells could be modified to carry different targeted therapies, for example sTRAIL, there may be an increase in the concentration delivered to LSCs present within the tumor.

Study of the role of the microenvironment in terms of LSC survival is very important; however the focus should be more on the communication between the microenvironmentand the stem cells. Until we learn more about the microenvironment's effect on regular HSCs, targeting of the communication or signaling pathways between them should be considered.

## Conclusions

The overall goal of cancer therapy is to target the cancer cells only while leaving viable normal cells unscathed. Some types of cancers can make this job seem impossible given the inability to distinguish. However, with current evidence, the future of targeted CSC eradication does not seem like such a daunting task. Although targeting of CSCs by their specific cell surface markers seems like a very logical approach to target therapy, results seem to indicate that other targeting strategies like signaling pathways or microenvironment may offer better results. This is not to say that identification of cell surface markers does not have its place in terms of studying CSCs. If we can identify different populations of cells that exhibit these cell surface markers and identify them as stem cells we can evaluate the effectiveness of new targeting strategies on that population.

By targeting other characteristics of stem cells, such as specific pathways they use or ways they manipulate their environment for survival, benefit can be seen without spending time on research to prove the hypothesis of CSCs. If we can show now that there are cells that have specific behaviors that decrease apoptosis or efflux mechanisms that make them resistant, then we are one step further in finding treatment to destroy them regardless of whether they are CSCs or cells that have obtained certain survival mechanisms through evolution. Table 2 summarizes ongoing clinical trials targeting CSC[44].

Increasing research is being aimed at targeting CSCs as opposed to the conventional targeting of homologous tumor cells. With increasing evidence, an intricate puzzle is being pieced together that is revealing an image consistent with targeted CSC therapy using those unique CSC probes in a nanotechnology-based targeted delivery with cytotoxic agents of CSC and cancer cells. In conclusion, targeting CSC, cancer cells, and its associated micro-environment might provide novel strategies in the management of cancer. However, there is a critical need for more direct surrogate markers (imaging of CSC reduction in the tumor microenvironment or reduction of circulating CSC) to assess the direct impact of those CSC targeted therapies in clinical trials listed in Table 2.

## Competing interests

The authors declare that they have no competing interests.

## Authors' contributions

SC and SM have equally contributed to the elaboration of the review. SM was the senior author. All authors read and approved the final manuscript.

## References

[B1] Bonnet D Dick JE Human acute myeloid leukemia is organized as a hierarchy that originates from a primitive hematopoietic cell Nat Med 1997 3 7 730 7 10.1038/nm0797-730 9212098

[B2] Tang C Ang BT Pervaiz S Cancer stem cell: target for anti-cancer therapy FASEB J 2007 21 14 3777 85 10.1096/fj.07-8560rev 17625071

[B3] Karp JE ed Acute Myelogenous Leukemia 2007 Humana Press: Totowa

[B4] ten Cate B Targeted elimination of leukemia stem cells; a new therapeutic approach in hemato-oncology Curr Drug Targets 2010 11 1 95 110 10.2174/138945010790031063 20017722

[B5] ten Cate B A novel AML-selective TRAIL fusion protein that is superior to Gemtuzumab Ozogamicin in terms of in vitro selectivity, activity and stability Leukemia 2009 23 8 1389 97 10.1038/leu.2009.34 19262596

[B6] Milligan DW Guidelines on the management of acute myeloid leukaemia in adults Br J Haematol 2006 135 4 450 74 10.1111/j.1365-2141.2006.06314.x 17054678

[B7] Leith CP Acute myeloid leukemia in the elderly: assessment of multidrug resistance (MDR1) and cytogenetics distinguishes biologic subgroups with remarkably distinct responses to standard chemotherapy. A Southwest Oncology Group study Blood 1997 89 9 3323 9 9129038

[B8] Sievers EL Selective ablation of acute myeloid leukemia using antibody-targeted chemotherapy: a phase I study of an anti-CD33 calicheamicin immunoconjugate Blood 1999 93 11 3678 84 10339474

[B9] Sievers EL Efficacy and safety of gemtuzumab ozogamicin in patients with CD33-positive acute myeloid leukemia in first relapse J Clin Oncol 2001 19 13 3244 54 11432892 10.1200/JCO.2001.19.13.3244

[B10] Larson RA Final report of the efficacy and safety of gemtuzumab ozogamicin (Mylotarg) in patients with CD33-positive acute myeloid leukemia in first recurrence Cancer 2005 104 7 1442 52 10.1002/cncr.21326 16116598

[B11] Lowenberg B Gemtuzumab ozogamicin as postremission treatment in AML at 60 years of age or more: results of a multicenter phase 3 study Blood 2010 115 13 2586 91 10.1182/blood-2009-10-246470 20103782

[B12] Walter RB CD33 expression and P-glycoprotein-mediated drug efflux inversely correlate and predict clinical outcome in patients with acute myeloid leukemia treated with gemtuzumab ozogamicin monotherapy Blood 2007 109 10 4168 70 10.1182/blood-2006-09-047399 17227830 PMC1885511

[B13] Jawad M Analysis of factors that affect in vitro chemosensitivity of leukaemic stem and progenitor cells to gemtuzumab ozogamicin (Mylotarg) in acute myeloid leukaemia Leukemia 2010 24 1 74 80 10.1038/leu.2009.199 19776761

[B14] Zhao X Targeting C-type lectin-like molecule-1 for antibody-mediated immunotherapy in acute myeloid leukemia Haematologica 2010 95 1 71 8 10.3324/haematol.2009.009811 19648166 PMC2805740

[B15] van Rhenen A The novel AML stem cell associated antigen CLL-1 aids in discrimination between normal and leukemic stem cells Blood 2007 110 7 2659 66 10.1182/blood-2007-03-083048 17609428

[B16] Tang R Zosuquidar restores drug sensitivity in P-glycoprotein expressing acute myeloid leukemia (AML) BMC Cancer 2008 8 51 10.1186/1471-2407-8-51 18271955 PMC2258302

[B17] Latagliata R Liposomal daunorubicin versus standard daunorubicin: long term follow-up of the GIMEMA GSI 103 AMLE randomized trial in patients older than 60 years with acute myelogenous leukaemia Br J Haematol 2008 143 5 681 9 10.1111/j.1365-2141.2008.07400.x 18950458

[B18] Robert G Fenton DLL Cancer Cell Biology and Angiogenesis 2008 The McGraw-Hill Companies, Inc

[B19] Martelli AM The phosphatidylinositol 3-kinase/AKT/mammalian target of rapamycin signaling network and the control of normal myelopoiesis Histol Histopathol 2010 25 5 669 80 20238304 10.14670/HH-25.669

[B20] Martelli AM Targeting the PI3K/AKT/mTOR signaling network in acute myelogenous leukemia Expert Opin Investig Drugs 2009 18 9 1333 49 10.1517/14728220903136775 19678801

[B21] Sirolimus

[B22] Bleau AM PTEN/PI3K/Akt pathway regulates the side population phenotype and ABCG2 activity in glioma tumor stem-like cells Cell Stem Cell 2009 4 3 226 35 10.1016/j.stem.2009.01.007 19265662 PMC3688060

[B23] Goodell MA Isolation and functional properties of murine hematopoietic stem cells that are replicating in vivo J Exp Med 1996 183 4 1797 806 10.1084/jem.183.4.1797 8666936 PMC2192511

[B24] Bapat S ed Cancer Stem Cells: Identification and Targets 2009 John Wiley & Sons: Hoboken 29 45 73-81

[B25] Yuan TL Cantley LC PI3K pathway alterations in cancer: variations on a theme Oncogene 2008 27 41 5497 510 10.1038/onc.2008.245 18794884 PMC3398461

[B26] Muranyi AL Dedhar S Hogge DE Targeting integrin linked kinase and FMS-like tyrosine kinase-3 is cytotoxic to acute myeloid leukemia stem cells but spares normal progenitors Leuk Res 2010 34 10 1358 65 10.1016/j.leukres.2010.01.006 20193963

[B27] Courtney KD Corcoran RB Engelman JA The PI3K pathway as drug target in human cancer J Clin Oncol 2010 28 6 1075 83 10.1200/JCO.2009.25.3641 20085938 PMC2834432

[B28] Hess G Phase III study to evaluate temsirolimus compared with investigator's choice therapy for the treatment of relapsed or refractory mantle cell lymphoma J Clin Oncol 2009 27 23 3822 9 10.1200/JCO.2008.20.7977 19581539

[B29] Hainsworth JD Phase II Trial of Bevacizumab and Everolimus in Patients With Advanced Renal Cell Carcinoma J Clin Oncol 2010 28 13 2131 6 10.1200/JCO.2009.26.3152 20368560

[B30] Ghobrial IM Phase II trial of the oral mammalian target of rapamycin inhibitor everolimus in relapsed or refractory Waldenstrom macroglobulinemia J Clin Oncol 2010 28 8 1408 14 10.1200/JCO.2009.24.0994 20142598 PMC2834498

[B31] Pandolfi PP Breast cancer--loss of PTEN predicts resistance to treatment N Engl J Med 2004 351 22 2337 8 10.1056/NEJMcibr043143 15564551

[B32] Peng C PTEN is a tumor suppressor in CML stem cells and BCR-ABL-induced leukemias in mice Blood 2010 115 3 626 35 10.1182/blood-2009-06-228130 19965668 PMC2810991

[B33] Von Hoff DD Inhibition of the hedgehog pathway in advanced basal-cell carcinoma N Engl J Med 2009 361 12 1164 72 10.1056/NEJMoa0905360 19726763

[B34] Rudin CM Treatment of medulloblastoma with hedgehog pathway inhibitor GDC-0449 N Engl J Med 2009 361 12 1173 8 10.1056/NEJMoa0902903 19726761 PMC5317279

[B35] Hofmann I Hedgehog signaling is dispensable for adult murine hematopoietic stem cell function and hematopoiesis Cell Stem Cell 2009 4 6 559 67 10.1016/j.stem.2009.03.016 19497284 PMC3065323

[B36] Fang J Seki T Maeda H Therapeutic strategies by modulating oxygen stress in cancer and inflammation Adv Drug Deliv Rev 2009 61 4 290 302 10.1016/j.addr.2009.02.005 19249331

[B37] Kotamraju S Williams CL Kalyanaraman B Statin-induced breast cancer cell death: role of inducible nitric oxide and arginase-dependent pathways Cancer Res 2007 67 15 7386 94 10.1158/0008-5472.CAN-07-0993 17671209

[B38] Chen R Combination of simvastatin and imatinib sensitizes the CD34+ cells in K562 to cell death Med Oncol 2010 10.1007/s12032-010-9472-9 20354828

[B39] Li R P-glycoprotein antibody functionalized carbon nanotube overcomes the multidrug resistance of human leukemia cells ACS Nano 2010 4 3 1399 408 10.1021/nn9011225 20148593

[B40] Li GY Cyclosporine diminishes multidrug resistance in K562/ADM cells and improves complete remission in patients with acute myeloid leukemia Biomed Pharmacother 2009 63 8 566 70 10.1016/j.biopha.2008.10.008 19095404

[B41] Balyasnikova IV Mesenchymal stem cells modified with a single-chain antibody against EGFRvIII successfully inhibit the growth of human xenograft malignant glioma PLoS One 2010 5 3 e9750 10.1371/journal.pone.0009750 20305783 PMC2841188

[B42] Wei Z Bone marrow mesenchymal stem cells from leukemia patients inhibit growth and apoptosis in serum-deprived K562 cells J Exp Clin Cancer Res 2009 28 141 10.1186/1756-9966-28-141 19883517 PMC2779804

[B43] Mishima K Growth suppression of intracranial xenografted glioblastomas overexpressing mutant epidermal growth factor receptors by systemic administration of monoclonal antibody (mAb) 806, a novel monoclonal antibody directed to the receptor Cancer Res 2001 61 14 5349 54 11454673

[B44] Morrison R Targeting the mechanisms of resistance to chemotherapy and radiotherapy with the cancer stem cell hypothesis J Oncol 2011 2011 941876 20981352 10.1155/2011/941876 PMC2958340

